# Alpha-1 blocker use increased risk of subsequent renal cell carcinoma: A nationwide population-based study in Taiwan

**DOI:** 10.1371/journal.pone.0242429

**Published:** 2020-11-19

**Authors:** Shian-Ying Sung, Trang Thi Huynh Le, Jin- Hua Chen, Teng-Fu Hsieh, Chia-Ling Hsieh

**Affiliations:** 1 The Ph.D. Program for Translational Medicine, College of Medical Science and Technology, Taipei Medical University, Taipei, Taiwan; 2 International Ph.D. Program for Translational Science, College of Medical Science and Technology, Taipei Medical University, Taipei, Taiwan; 3 TMU Research Center of Cancer Translational Medicine, Taipei Medical University, Taipei, Taiwan; 4 Office of Human Research, Taipei Medical University, Taipei, Taiwan; 5 International Master/Ph.D. Program in Medicine, College of Medicine, Taipei Medical University, Taipei, Taiwan; 6 Graduate Institute of Data Science, College of Management, Taipei Medical University, Taipei, Taiwan; 7 Research Center of Biostatistics, College of Management, Taipei Medical University, Taipei, Taiwan; 8 Biostatistics Center, Wan Fang Hospital, Taipei Medical University, Taipei, Taiwan; 9 Department of Urology, Taichung Tzu Chi Hospital, Buddhist Tzu Chi Medical Foundation, Taichung, Taiwan; University Medical Center Utrecht, NETHERLANDS

## Abstract

Elevated Renal cell carcinoma (RCC) risk has been associated with the use of several antihypertensive medications but has not yet been elucidated in the populations prescribed alpha-1 blockers that are commonly used in the treatment of hypertension and lower urinary tract symptoms associated with benign prostatic hyperplasia (LUTS-BPH). The aim of the present study was to investigate the association between alpha-1 blocker use and the risk of developing RCC using a nationwide population-based database in Taiwan. Patients who were treated with alpha-1 blockers for at least 28 days were identified through the Taiwan National Health Insurance Research Database from 2000 to 2010. The unexposed participants were matched with the exposed cases according to age, sex, and index year at a ratio of 3:1. Cox proportional hazards regression, stratified by sex and comorbidities and adjusted for age, was performed to estimate hazard ratios (HRs) for the risk of subsequent RCC. Among 2,232,092 subjects, patients who received alpha-1 blocker treatment had a higher risk of RCC than the unexposed group. Taking into account hypertension and BPH, the adjusted HR was significantly higher in male alpha-1 blocker users who had no BPH and either the presence (HR: 1.63, 95% confidence interval [CI] = 1.22–2.18) or absence (HR: 2.31, 95% CI = 1.40–3.81) of hypertension than in men not receiving these drugs. Taken together, male alpha-1 blocker users who had no comorbidity of BPH exhibited an increased risk for developing RCC independent of hypertension. Further study is warranted to elucidate the underlying mechanisms of this association.

## Introduction

Renal cell carcinoma (RCC) is the most common malignant form of kidney cancer that arises from the renal epithelium. RCC accounts for 5% and 3% of all oncological diagnoses in men and in women, respectively [1] and is an often lethal malignancy for which there is no effective preventative and therapeutic strategy. Age and sex are strongly related to the risk of RCC. Overall, the incidence of RCC is approximately 1.6 to 2.0 times higher in men than in women after adjustment for age, and the estimated incidence increases in the older population, with a peak between 60 and 70 years of age [2]. Hypertension is also a risk factor for RCC; the relationship was identified in several large prospective cohort studies [3, 4]. In this regard, effective blood pressure control may lower the risk of RCC. However, several retrospective observational studies have found a link between elevated RCC risk and the use of antihypertensive medications, including diuretics, calcium channel blockers, beta-blockers, and angiotensin-converting enzyme inhibitors [5–8], although it is unclear whether the increased risk is caused by hypertension itself or by the use of antihypertensive medication.

Alpha-1 adrenoceptor antagonists (also called alpha-1 blockers) are a family of agents that cause a decrease in total peripheral resistance and are efficacious in the management of hypertension when used alone or in combination with other medications. Mechanistically, these drugs inhibit the effects of norepinephrine on the alpha-1 subtype of adrenergic receptors (α1-ARs) in blood vessels to promote vasodilation [9], and they also directly targets the smooth muscle cells in the prostate gland and bladder neck resulting in a decrease in smooth muscle tone and relief of bladder outflow obstruction [10]. Thus, alpha-1 blockers, although originally developed for the treatment of arterial hypertension, have become the first line of pharmacologic management for lower urinary tract symptoms in men with benign prostatic hyperplasia (LUTS-BPH) [11–13].

In addition to the traditional actions on smooth muscle relaxation and decrease in blood pressure, emerging studies have demonstrated the potentiation of alpha-1 blockers in growth inhibition, apoptosis, and angiogenesis suppression in an α1-adrenoceptor-independent manner. The potential mechanisms, including the inactivation of CDK [14], disruption of DNA integrity [15], engaging death receptor-mediated caspase-8 activation [16], induction of transforming growth factor-β signaling [17], downregulation of vascular endothelial growth factor [18, 19], and targeting focal adhesion survival signaling [20], have provided molecular underpinnings for the pharmacological exploitation of alpha-1 blocker therapy for a range of epithelial cancers, such as advanced prostate cancer, bladder cancer, and RCC [21].

Because of the increasing prevalence of BPH and hypertension with the increase in the mean age of the population, the use of alpha-1 blockers has steadily increased [22]. It is important to determine whether alpha-1 blockers could increase RCC incidence similarly to other reported antihypertensive drugs or conversely if they could decrease the risk through the proposed anticancer activity under current clinical practice. In this study, we aimed to investigate the relationship between alpha-1 blocker use and the risk of RCC using the National Health Insurance Research Database (NHIRD), a population-based database derived from the claims data of the National Health Insurance program of Taiwan [23].

## Materials and methods

### Data source

Data for this study were obtained from the NHIRD, which is derived from Taiwan’s National Health Insurance program and is currently maintained and regulated by the Data Science Center of the Taiwan Ministry of Health and Welfare for public research purposes. The NHIRD is one of the largest administrative healthcare databases in the world, containing comprehensive clinical information of over 99% of Taiwan’s residents (approximately 23 million population) who were enrolled in this program. The datasets of NHIRD contain detailed healthcare information on demographics as well as all medical claims data, including patient demographics, medical visits, laboratory tests, procedures, prescriptions, and primary and secondary diagnoses based on the International Classification of Diseases, Ninth Revision, Clinical Modification (ICD-9-CM) coding. The information in the NHIRD is stored in different datasheets; to protect patient privacy, data are de-identified secondary data [23].

### Study population

We identified a cohort of patients with exposure to alpha-1 blocker medication in NHIRD on the basis of the presence of a prescription/dispensing record for at least 28 consecutive days in the study period from 1 January 2000 to 31 December 2010 (Accessed: July 2016). The 28th day after the date of the first prescription was set as the exposure index date. Eligible study subjects were those aged ≤90 years on the exposure index date who had not received the drug in the past 1 year (defined as new users). Patients who had a diagnostic record of malignancy, either RCC or any type of cancer, before or within 365 days after the exposure index date as identified using ICD-9-CM codes from the Registry for Catastrophic Illness Patients (RCIP) in the NHIRD were excluded from this study. The unexposed group consisted of patients who did not have a prescription record of alpha-1 blockers in the study period. The unexposed participants were matched with the exposed patients according to age, sex, and index year at a ratio of 3:1. Control patients were assigned the same index date as their matched cases. The unexposed cohort was subject to the same data quality checks and exclusions as the exposed cohort.

### Exposure drugs

Data on all medications were collected using the Anatomical Therapeutic Chemical Classification system codes of the World Health Organization. The alpha-1 blockers investigated in this study included doxazosin (A043765100, A044681100, A044853100, A050263100, AC42378100, AC42874100, AC43467100, AC44567100, AC44621100 AC45013100; 2–8 mg/day), prazosin (AC26741100, AC267411G0, AC30415100, and AC39962100, B021220100, B021221100, B0212211G0; 1–8 mg/day), terazosin (A042936100, A043188100, A043317100, A043625100, A043656100, A043961100, AC44063100, AC44547100, B017664100; 2–10 mg/day), and tamsulosin (AB46016100, B024403100, B025413100; 0.2–0.8 mg/day).

### Comorbidities and outcome

Comorbid medical disorders, including hypertension and BPH, before the exposure index date were investigated as the baseline comorbidities on the basis of all available history records in the NHIRD using the diagnosis codes ICD-9-CM 401–405 and ICD-9-CM 600, respectively. The outcome was a new diagnosis of RCC (ICD-9-CM: 189.0) given at a psychiatric contact (as in‐patients or out‐patients) and as identified in the RCIP after at least 365 days from the exposure index date.

### Study follow-up

All cases of this study were followed up for the presence of incident RCC (ICD-9-CM: 189.0) over time until the occurrence of RCC or the end of the study period (December 31, 2010).

### Statistical analysis

Standard descriptive methods were used to describe demographic and clinical characteristics and comorbid medical disorders for the total population, given as counts and corresponding percentages or as the mean and corresponding standard deviation. The incidence rate of RCC was measured using person-time of the total cohort follow-up and presented as a percentage or number per 100,000 person-years. Cox regression models were used to determine the associations between alpha-1 blocker use and RCC incidence by analyzing hazard ratios (HRs) and 95% confidence intervals (95% CIs). The multivariable analysis was adjusted for the predefined variables age, sex, Charlson comorbidity index (CCI) score, and comorbidities (hypertension and BPH) in the regression model. We also used Cox proportional hazards regression models to assess the interaction between alpha-1 blockers and comorbidities for incident RCC. The chi-square test was used to determine the relationship between different variables. Data management and analyses were performed using SAS 9.4 software (SAS Institute Inc., Cary, NC, USA). *P* < .01 was considered statistically significant.

### Ethics approval and consent to participate

This study was approved by the Taipei Medical University Research Ethics Committee (TMU-JIRB N201612053). Informed consent was waived because this was a retrospective study using NHIRD data.

## Results

### Characteristics of study participants

**Fig 1:**
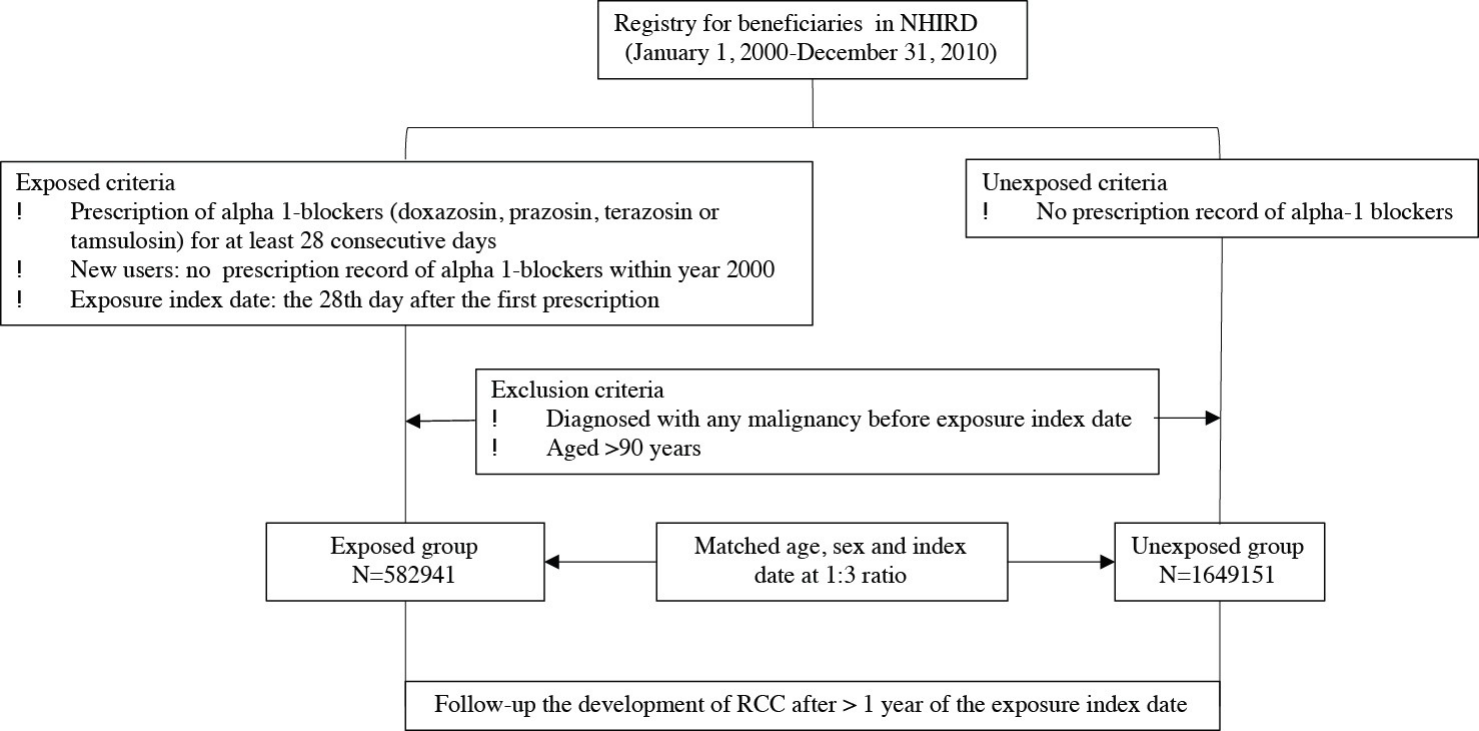
Flowchart of participants with and without alpha-1 blockers treatment recruited from NHIRD in Taiwan during 2000–2010.

A flowchart of the study selection process is shown in Fig 1. In a sampling cohort of approximately 2.3 million participants enrolling between January 1, 2000 and December 31, 2010, a total of 582,941 patients were identified as new alpha-1 blocker users and classified as exposed after exclusion of those who were prescribed alpha-1 blockers in 2000, diagnosed as having RCC and other cancers prior to index day, and aged > 90 years. Another 1,649,151 patients, frequency-matched by age (every 5 years), sex, and index year of exposed subjects, were identified as the unexposed control group.

The baseline demographic data for the cohort are summarized in Table 1. Most participants in the alpha-1 blocker–exposed group were men (79.5%); the mean age was 65.26 ± 14.61 years. The unexposed control group was well matched for sex and age. Alpha-1 blockers are established medications for treating hypertension and LUTS-BPH [11–13]. As expected, a larger population of participants with comorbidities of hypertension and BPH was observed in the exposed group than in the control group (63.7% vs. 28.8% and 43.9% vs. 1.6%, respectively).

**Table 1:** Baseline characteristics of study participants after the matching procedure.

Variable	α1- blocker			
	Unexposed (N = 1,649,151)		Exposed (N = 582,941)	
	n	%	n	%
Gender				
Male	1,289,265	79.2	463,702	79.5
Female	359,886	20.8	119,239	20.5
Age, mean (SD)	65.23	(14.68)	65.26	(14.61)
Comorbidities				
Hypertension	475,391	28.8	371,087	63.7
BPH	25,820	1.6	255,735	43.9
Outcome				
RCC (per 100,000 person-years)	359	(3.90)	289	(8.91)

Among all 2,232,092 enrolled patients, 648 developed RCC during the study’s follow-up period. The unexposed and exposed groups had 359 and 289 patients with RCC, respectively. The prevalence rates of RCC were 3.90 per 100,000 person-years in the unexposed group and 8.91 per 100,000 person-years in the exposed group.

### Association of the alpha-1 blockers exposed cohort with an increased risk of RCC

Table 2 shows the results of the multivariable analysis of the incidence of RCC. The patients in the alpha-1 blocker–exposed group had a significantly higher crude HR of 2.28 (95% CI, 1.96−2.67; *P* < .001) than those in the unexposed control group. This association remained substantially unchanged (adjusted HR, 1.71; 95% CI, 1.402−2.091; *P* < .001) even after adjustment for potential confounders, including age, sex, CCI, and comorbidities of hypertension and BPH (adjusted model). Furthermore, hypertension was another risk factor for RCC (HR, 1.859; 95% CI, 1.553−2.226; *P* < .001), whereas age, sex, CCI score, and BPH were not the significant variables influencing RCC incidence.

**Table 2:** Crude and adjusted hazard ratios of RCC using Cox proportional hazard regression model.

Characteristics	Hazard ratios (95% confidence interval)			
	Crude	P-value	Adjusted model†	P-value
Exposed group	2.28 (1.96–2.67)	<0.001*	1.710 (1.402–2.091)	<0.001*
Age group (ref = 0–50 y)			0.998 (0.992–1.004)	0.529
Sex (ref = women)			1.048 (0.864–1.271)	0.663
CCI score			0.990 (0.930–1.053)	0.740
Hypertension (ref = none)			1.859 (1.553–2.226)	<0.001*
BPH (ref = none)			1.168 (0.918–1.487)	0.206
Control	1.00		1.00	

* *P* < 0.01

** *P* < 0.001 (Chi-square test)

† Adjusted model was adjusted for age, gender, CCI, and comorbidity of hypertension and BPH

Because hypertension and BPH were related to alpha-1 blocker use, we also performed a stratified analysis to determine whether these disease conditions influenced the effect of alpha-1 blockers on RCC. In this way (Table 3), an increased risk for RCC among alpha-1 blocker users was observed in the subgroup of patients without BPH (adjusted HR, 1.64; 95% CI, 1.33−2.22; *P* < .001) but not in those with BPH (adjusted HR, 0.95; 95% CI, 0.51−1.17; *P* < .001). Nonetheless, the adjusted HR of RCC was still higher in the alpha-1 blocker–exposed cohort, regardless of whether the patients were diagnosed as having hypertension (adjusted HR, 1.53; 95% CI, 1.21−1.93; *P* < .001 for the presence of hypertension; odds ratio, 1.06; 95% CI, 1.04−2.45; *P* < .05 for the absence of hypertension).

**Table 3:** Subgroup analysis of the risk of RCC between exposed and unexposed groups according to sex and presence or absence of hypertension and BPH.

Subgroup	No. of RCC	Adjusted HR (95% CI) †
alphal-1 blockers with BPH		
Exposed	116	0.95 (0.51–1.17)
Unexposed	11	1.00
alphal-1 blockers without BPH		
Exposed	173	1.64 (1.33–2.02)**
Unexposed	348	1.00
alphal-1 blockers with hypertension		
Exposed	204	1.53 (1.21–1.93)**
Unexposed	157	1.00
alphal-1 blockers without hypertension		
Exposed	85	1.60 (1.04–2.45)*
Unexposed	202	1.00

† Adjusted for age, gender, CCI, hypertension and BPH.

* *P* < 0.01

** *P* < 0.001 (Chi-square test).

### Interaction of hypertension and BPH for association of alpha-1 blocker use and RCC risk

When further stratified by sex (Table 4), a strong association between alpha-1 blocker use and RCC was found in men (HR, 1.89; 95% CI, 1.49−2.39) but not in women (HR, 1.44; 95% CI, 0.99−2.11). In addition, among male participants, alpha-1 blocker users with hypertension conferred a significantly increased risk for RCC only when the patients did not have BPH (HR, 1.63; 95% CI, 1.22−2.18; *P* < .001). However, the association of alpha-1 blocker exposure and RCC risk seen in patients without BPH was still high in male populations, not only in the subgroup with hypertension but also in the one without hypertension (HR, 2.31; 95% CI, 1.40−3.81; *P* < .001). Notably, although women do not have a prostate and never develop BPH, no statistically significant interaction was found for hypertension with the association of alpha-1 blocker use and RCC incidence in women.

**Table 4:** Stratified analysis of the adjusted hazard ratios of α-1 blockers for RCC by gender and comorbidity of hypertension and BPH.

Characteristics		No. of RCC (Exposed vs. Unexposed)	Adjusted HR (95% CI) †	
			α-1 blockers	
			Exposed	Unexposed
Men				
	All	299 vs. 268	1.89 (1.21–1.93)*	1 (Ref)
	Non-BPH + Non-hypertension	18 vs. 148	2.31 (1.40–3.81)*	1 (Ref)
	BPH + Non-hypertension	63 vs. 5	1.05 (0.42–2.63)	1 (Ref)
	Non-BPH + hypertension	95 vs. 109	1.63 (1.22–2.18)*	1 (Ref)
	BPH + hypertension	53 vs. 6	0.85 (0.36–2.00)	1 (Ref)
Women				
	All	60 vs. 91	1.44 (0.99–2.11)	1 (Ref)
	Non-hypertension	4 vs. 49	1.06 (0.37–3.06)	1 (Ref)
	hypertension	56 vs. 42	1.48 (0.97–2.26)	1 (Ref)

* Chi-square test. Significance at *P* < 0.01

† Adjusted for age and CCI.

Taken together, a strong association was observed between alpha-1 blocker use and the risk of RCC in the male population, with the highest HR in those with neither hypertension nor BPH compared with the unexposed men.

## Discussion/conclusion

To our knowledge, this study is the first to determine the relationship between exposure to alpha-1 blockers and RCC incidence. This retrospective analysis provides the first epidemiologic evidence of the association of alpha-1 blocker use with an increased risk of RCC. Among men without a history of BPH, RCC was greatly associated with long-term use (>28 days) of alpha-1 blockers, whereas women showed no association after confounders were controlled for in the analysis. Although indications for alpha-1 blockers are LUTS-BPH and controlling blood pressure adequately for patients with hypertension, in Taiwan, physicians prescribed alpha-1 blockers also for other purposes, such as medical expulsive therapy for urolithiasis, treatment of neuropathic voiding dysfunction, improvement of bladder emptying from dysfunctional voiding and urinary retention, and reduction of total serum cholesterol level [24–26]. Thus, the recommendation of the present study to explore the risk of developing RCC after alpha-1 blocker use raises awareness among clinicians and public health authorities of the potential consequences of long-term use of alpha-1 blockers for indications other than LUTS-BPH.

A strength of our study is that it was a nationwide population-based study rather than involving a self-reported database. The NHIRD has nearly complete follow-up information regarding healthcare use by all study participants, and the dataset is routinely monitored. Considering the differences in age, sex, and comorbidities between the patients who did and did not receive alpha-1 blockers, propensity score matching was applied to select the control subjects. Nonetheless, we cannot exclude the limitations of the retrospective design, which completely relied on ICD-9-CM coding data for classifying BPH and hypertension. Even though the NHI Bureau of Taiwan regularly performs audits on the validity of diagnosis, we did not evaluate the accuracy of RCC ICD-9-CM codes in the secondary position. In addition, established risk factors for RCC, such as obesity, tobacco use, family history of kidney cancer, and certain dietary factors, were not taken into consideration, owing to a lack of sufficient information conveyed in the codes. However, given the magnitude and statistical significance of the observed effects in this study, these limitations are unlikely to have compromised the results.

Only a few clinical studies have examined the relationship between alpha-1 blockers and cancer risk, and the results remain controversial. Researchers in a 5-year retrospective cohort study at Lexington Veterans Affairs Medical Center observed that men exposed to quinazoline-derived alpha-blockers (doxazosin, prazosin, and terazosin) were at a 1.46 times lower relative risk and 31.7% lower attributable risk for prostate cancer than untreated patients [27]. Another retrospective 20‐year cohort study of men residing in Saskatchewan demonstrated that users of alpha-1 blockers, including alfuzosin, doxazosin, prazosin, tamsulosin, or terazosin, had an 11% lower risk of a prostate cancer diagnosis than nonusers [28]. However, in the Finnish Prostate Cancer Screening Trial, use of tamsulosin and alfuzosin had no effect on overall risk of prostate cancer [29]. Moreover, the positive association of prescribed alpha-1 blockers with prostate cancer risk was also observed in a nationwide cohort study in Denmark [30] and in a case–control analysis of a cohort study in Northern California [31]. Our present study investigating exposure to doxazosin, prazosin, terazosin and tamsulosin implies that alpha-1 blockers increase RCC incidence in men. The conflicting findings on the relationship between alpha-1 blockers and cancer risk remains unclear but may relate in part to the distinct chemical structures of drugs. *In vitro* studies demonstrated that quinazoline-based antagonists, including doxazosin, terazosin, and prazosin, induced apoptosis in benign and malignant prostate cells via an alpha-1-adrenoceptor-independent action [32–35]. However, the sulfonamide derivative tamsulosin does not elicit similar cytotoxic activity against cancer cells [36]. More robust trials with standardized stratification for comparing different classes of alpha-1 blockers are crucial to verifying the hazard assessment results.

Mechanistically, alpha-1 blockers selectively antagonize ARs through a postsynaptic blockade and thus inhibit smooth muscle contraction. Molecular biological studies have clearly defined three α1-AR subtypes: α1A (formerly named α1C), α1B, and α1D. These subtypes can be pharmacologically distinguished by their differential binding to alpha-1 blockers and may be linked to distinct second-messenger systems [37]. In the kidney, α1A- and α1D-ARs are the major functional subtypes other than α1B [38]. A previous in vitro study on rat cell lines transfected with human α1-AR cDNAs demonstrated that α1A- and α1D-ARs mediate G_1_-S cell cycle arrest, whereas the α1B-ARs mediate cell cycle progression [39]. The differential regulation of cell growth has been further evidenced in transgenic mice. Although long-term α1B-AR activation had no effect on cancer, α1A-AR stimulation prolonged lifespan in association with decreased cancer incidence [40]. Moreover, tamsulosin has a 10-fold greater affinity for α1A- and α1D-ARs than for α1B-ARs [41]. Accordingly, it can be suspected that long-term blockage of α1A- and α1D-ARs by using alpha-1 blockers, in particular the α1A/α1D-AR-selective antagonist tamsulosin, may increase cancer risk, which may account in part for our present findings of a strong association of alpha-1 blocker use with RCC incidence. However, this explanation cannot clarify why the risk association exists only in men and not in women. Further elucidation of sex differences in relation to the α1-AR subtype distribution and functional response to alpha-1 blockers could lead to a better understanding of the underlying mechanisms.
